# An up-to-date review of rutin and its biological and pharmacological activities

**DOI:** 10.17179/excli2014-663

**Published:** 2015-01-09

**Authors:** Naif Abdullah Al-Dhabi, Mariadhas Valan Arasu, Chang Ha Park, Sang Un Park

**Affiliations:** 1 Department of Botany and Microbiology, Addiriyah Chair for Environmental Studies, College of Science, King Saud University, P. O. Box 2455, Riyadh 11451, Saudi Arabia; 2 Department of Crop Science, Chungnam National University, 99 Daehak-ro, Yuseong-gu, Daejeon, 305-764, Korea; 3 Visiting Professor Program (VPP), King Saud University, P.O. Box 2455, Riyadh 11451, Saudi Arabia

## Dear Editor

**Figure 1:**
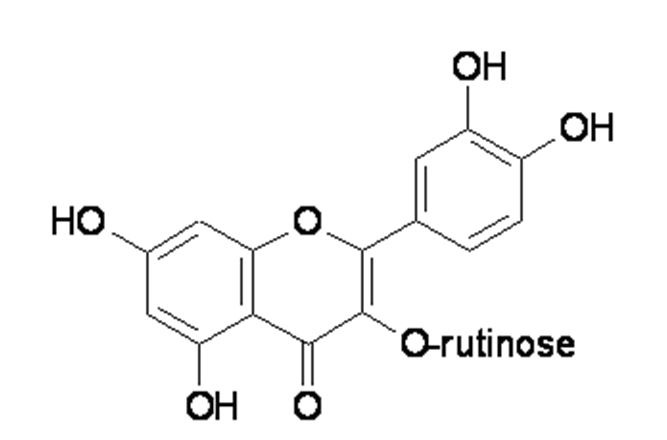
Chemical structure of rutin

Rutin (3,3',4',5,7-pentahydroxyflavone-3-rhamnoglucoside: Figure 1(Fig. 1)) is a flavonoid of the flavonol-type that is widespread in the plant kingdom (Hosseinzadeh and Nassiri-Asl, 2014[6]). Rutin is synthesized through the phenylpropanoid metabolic pathway, which involves the transformation of the amino acid phenylalanine to 4-coumaroyl-CoA. 4-coumaroyl-CoA can be combined with malonyl-coA to produce the true backbone of flavonoids. The biosynthetic pathway continues through a series of enzymatic modifications to produce rutin (Li et al., 2014[9]). 

Rutin has a wide range of pharmacological properties (e.g., antioxidative activity) that have been exploited in human medicine and nutrition. Conventionally, it is used as an antimicrobial, antifungal, and anti-allergic agent. However, current research has shown its multi-spectrum pharmacological benefits for the treatment of various chronic diseases, such as cancer, diabetes, hypertension, and hypercholesterolemia (Sharma et al., 2013[23]). Here, we summarize the key messages of recent studies performed on rutin and its biological and pharmacological activities (Table 1(Tab. 1)). 

Table 1 (references):

The present findings demonstrate the potential of rutin and quercetin in mitigating radiation-induced mortality and cytogenetic damage, which may be attributed to the scavenging of radiation-induced free radicals (Patil et al., 2014[19]).

Rutin can induce bone formation via the differentiation of human MG-63 osteosarcoma cells. This is the first study to demonstrate the effectiveness of rutin as an osteoblast stimulant (Park et al., 2014[18]).

Rutin may improve endothelial function by augmenting nitric oxide production in human endothelial cells (Ugusman et al., 2014[26]).

Rutin exhibited significant antidiabetic activity, presumably by inhibiting inflammatory cytokines, and improved the antioxidant and plasma lipid profiles in high fat diet + streptozotocin-induced type 2 diabetic model. Rutin may thus be useful as a diabetic modulator along with standard antidiabetic drugs (Niture et al., 2014[15]).

Rutin protects against the neurodegenerative effects of prion accumulation by increasing the production of neurotropic factors and inhibiting apoptotic pathway activation in neuronal cells. These results suggest that rutin may have clinical benefits for prion diseases and other neurodegenerative disorders (Na et al., 2014[12]).

Rutin exerts anti-inflammatory effects in ultraviolet B-irradiated mouse skin by inhibiting the expression of cyclooxygenase-2 and inducible nitric oxide synthase (Choi et al., 2014[4]).

Rutin has only a weak and short-term anticonvulsant potential. It appears to be safe for patients with epilepsy because it neither changes the activity of the studied anti-epileptic drugs nor produces any adverse effects (Nieoczym et al., 2014[14]).

Rutin has multi-target therapeutic potential for cognitive deficits associated with conditions involving chronic cerebral hypoperfusion, such as vascular dementia and Alzheimer's disease (Qu et al., 2014[21]).

The study demonstrates for the first time the protective role of two common flavonoids, quercetin and its glycone rutin, against high cholesterol diet (2%)-induced hepatotoxicity and inflammation (Sikder et al., 2014[24]).

Rutin treatment ameliorates the various impairments associated with physical fatigue in a forced swimming mouse model (Su et al., 2014[25]).

Rutin may serve as a potential agent for glycemic control through enhancement of insulin-dependent receptor kinase activity, thereby inducing the insulin signaling pathway, and thus causing increased glucose transporter 4 translocation and increased glucose uptake (Hsu et al., 2014[7]).

Rutin may have therapeutic potential for the treatment of neurodegenerative diseases associated with oxidative stress (Park et al., 2014[18]).

Rutin could be effective in reducing neurotoxicity, and its neuroprotective effect might be mediated via antioxidant activity (Motamedshariaty et al., 2014[11]).

Rutin is a promising agent for the treatment of Alzheimer's disease because of its antioxidant, anti-inflammatory, and ß-amyloid oligomer-reducing activities (Xu et al., 2014[27]).

Nitric oxide modulation could possibly be involved in the neuroprotective effects of rutin against head trauma-induced cognitive deficits, neuroinflammation, and apoptotic signaling cascade (Kumar et al., 2014[8]).

These results suggest that rutin may protect against spatial memory impairment induced by trimethyltin. Synaptophysin and the dopaminergic system may be involved in trimethyltin-induced neuronal damage in the hippocampus (Zhang et al., 2014[29]).

Rutin could be a candidate therapeutic agent for the treatment of various severe vascular inflammatory diseases via inhibition of the HMGB1 (high mobility group box 1) signaling pathway (Yoo et al., 2014[28]).

The anti-inflammatory activity of rutin might be due to its modulation of the expression of the ASC (apoptosis-associated speck-like protein) complex that mediates inflammation (Aruna et al., 2013[2]).

Although the stimulatory effect of rutin is a little weak, it may affect the absorption of organic anion-transporting polypeptide (OATP) 2B1 substrates because rutin is taken daily in foods and its intestinal concentration would reach the stimulatory range of OATP2B1 (Ogura et al., 2014[16]).

The results of this study highlight the risk of high salt consumption on cardiovascular health and the potent antioxidant and antihypertensive property of rutin and quercetin (Olaleye et al., 2013[17]).

These results indicate that rutin has potential anticonvulsant and antioxidative activities against oxidative stress in kainic acid-induced seizure in mice (Nassiri-Asl et al., 2013[13]).

Oral supplementation with rutin restores impaired baroreflex sensitivity and vascular reactivity in hypertensive rats by decreasing oxidative stress (Mendes-Junior et al., 2013[10]).

Rutin might be useful as an adjuvant in radioiodine therapy, since this flavonoid increases thyroid iodide uptake without greatly affecting thyroid function (Goncalves et al., 2013[5]).

Rutin exerted its hypouricemic action and renal function improvement by the regulation of renal organic ion transporters (Chen et al., 2013[3]).

The data showed that rutin clearly attenuated hexachlorobutadiene-induced nephrotoxicity and has the potential to be considered as a nephroprotective agent (Sadeghnia et al., 2013[22]).

Rutin exerts in vitro cytotoxic effects on SW480 (Human colon adenocarcinoma cell line) cells, induces in vivo antitumor effects, lacks toxic effects on mice bearing SW480 tumors, and exerts anti-angiogenic properties (Alonso-Castro et al., 2013[1]).

The present findings demonstrate the potential of rutin and quercetin in mitigating radiation-induced mortality, which may be attributed to an elevation in the antioxidant status and anti-lipid peroxidative potential (Patil et al., 2012[20]).

## Acknowledgements

This project was supported by King Saud University, Deanship of Scientific Research, Addiriyah Chair for Environmental Studies.

## Figures and Tables

**Table 1:**
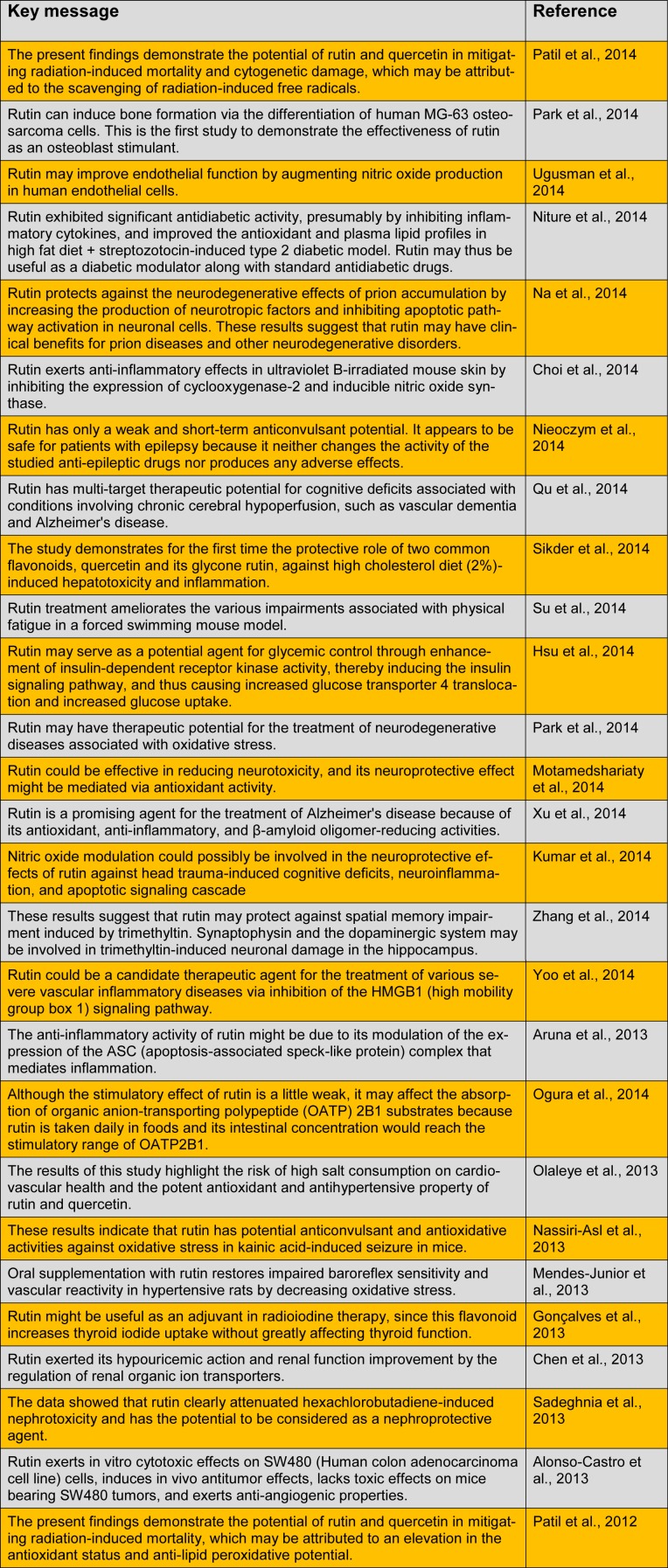
The biological and pharmacological activities of rutin, as revealed by recent studies
